# Selectivity of Leguminous Trees by Water Buffaloes in Semi-intensive Systems

**DOI:** 10.3389/fvets.2020.542338

**Published:** 2020-11-24

**Authors:** Maykel Andrés Galloso-Hernández, Vicente Rodríguez-Estévez, Carlos Armando Alvarez-Díaz, Mildrey Soca-Perez, Devon Ronald Dublin, Jesús Iglesias-Gómez, Leonel Simon Guelmes

**Affiliations:** ^1^Department of Animal Production, Universidad de Córdoba, Córdoba, Spain; ^2^Department of Basic Science, Universidad Técnica de Machala, Machala, Ecuador; ^3^Department of Research, Sustainable Systems of Animal Production, Experimental Station of Pastures and Forrage: Indio Hatuey, Matanzas, Cuba; ^4^Language Department, Hokkaido University of Education, Sapporo, Japan

**Keywords:** selectivity, feeding behavior, buffaloes (*Bubalus bubalis*), consumption, trees leaves, semi-intensive system

## Abstract

Water buffaloes (*Bubalus bubalis*) manifest different levels of selectivity for different pastures and forages. Knowledge of feed selectivity is important to facilitate the design of efficient production systems that take into account optimal animal welfare. In this study, the selectivity of nine 18-month old female water buffaloes for *Leucaena leucocephala, Albizia lebbeck, Gliricidia sepium*, and *Moringa oleifera* was evaluated. After 12 h of grazing *Megathyrsus maximum*, the animals were housed in individual shelters and 1.2 kg of leaves from each of the four tree species were offered to the animals simultaneously. The selectivity, measured as the intake of dry matter (DM), was highest for *A. lebbeck* (mean = 0.34 kgDM, SD = 0.05 kg), followed by *L. leucocephala* (mean = 0.30 kgDM, SD = 0.03 kg), *M. oleifera* (mean = 0.11 kgDM, SD = 0.05 kg), and *G. sepium* (mean = 0.10 kgDM, SD = 0.02 kg) (*P* < 0.01). The crude protein intake was highest for *A. lebbeck and L. leucocephala*. Notably, the less selected leaves were those of *G. sepium* and *M. oleifera*. This study suggests that the inclusion of *A. lebbeck* and *L. leucocephala* in silvopastoral systems may increase both the consumption and well-being of water buffaloes in the tropics.

## Introduction

The importance of buffaloes (*Bubalus bubalis*) as a productive species has increased worldwide by 2% in recent years with a total population of 202 million (1). For example, Cuba has a population of 6 × 10^4^ buffaloes (2, 3) with an annual increase of 13.7% and comparatively less than Brazil with 3 × 10^6^ buffaloes (4). In Argentina, the population amounts to 8.5 × 10^4^ with an annual growth of 13.1% (5).

The advantages of using buffaloes in tropical environments are the resistance to heat stress under shade (6, 7), and the fact that it allows this species to maintain birth rates above 80%, which is higher than cattle in similar conditions (8). However, the milk production per hectare (ha) is lower than cattle due to the low stocking rates (0.6–0.8 animals/ha) currently used to breed buffaloes (8, 9). The capacity of buffaloes to digest highly fibrous diets (10, 11) makes the use of trees an attractive strategy for the incorporation of proteins in the diet of these animals (12–15).

Tropical tree species are included in pastures in the form of silvopastoral systems. The incorporation of *Leucaena leucocephala* (13), *Albizia lebbeck* (16), *Gliricidia sepium*, and *Moringa oleifera* (17, 18) in silvopastoral systems improves the quality of feed for ruminants and decreases the impact of environmental stressors on the animals (6). The use of trees as a source of feed for ruminants has been ignored due to the limited understanding of their positive impact on production systems (19). Leguminous trees are a source of feed in sustainable livestock farming in the dry season (19–22). A good selection of trees maximizes the benefits of agroforestry systems (13, 21, 24). Leguminous trees (*L. leucocephala, A. lebbeck, G. sepium, and M. oleifera*) have perennial leaves and fruits with high nutritional value. Ruminal evaluations showed that diets comprised of pasture and tree leaves have a higher rate of degradation (i.e., organic material, dry matter, crude protein, gross energy). Leucaena trees, for example, have the highest rumen by-pass protein supply. This digestibility of the protein is 63% in *L. leucocephala* and around 60% in other tree species (25–28), which decreases the amount of commercial concentrates being used (21, 29, 30). According to Ku Vera et al. (26), we need to deepen our understanding through the manipulation of different diets in ruminants including the use of leguminous trees and grasses in tropical conditions. The branches of leguminous trees also contain condensed tannins with antiparasitic properties (31) that could reduce enteric CH4 emissions (30).

The productivity of buffaloes has been evaluated in silvopastoral systems in tropical countries including Cuba (8, 22, 33), and Brazil (34). Silvopastoral systems, combined with rusticity and high productive indexes (12, 21), improve the productive performance of buffaloes per ha by increasing the daily weight gain and milk production when compared to conventional systems (35). Previous studies have tested the effect of individual leguminous trees on ruminant metabolism (12, 25, 36). However, in natural conditions, animals select a mixture of foliage from different trees. The aim of this study was to evaluate the selectivity of water buffaloes for four tree species (*L. leucocephala, A. lebbeck, G. sepium, M. oleifera*) that are commonly used in silvopastoral systems as demonstrated in the aforementioned studies.

## Materials and Methods

### Ethics Statement

The experiment received the approval of the Scientific Council and the Ethics Committee of the “Indio Hatuey” Grass and Forage Experimental Station, Matanzas, Cuba. This was an observational study that did not involve any harm or cruelty to animals.

### Animals and Study Site

Clinically healthy 18-month old female water buffaloes (*n* = 9), with an average body weight of 373.77 kg, and daily weight gain of 362 g were used in the study. Before being included in the experiment, the animals were treated with ivermectin (1 μg/kg, Labiomec, Labiofam, Habana, Cuba) to ensure that their consumption behavior is not affected by intestinal parasites.

The study was conducted between August and September of 2008 in the municipality of Périco, Matanzas, Cuba, located at 22°48′7″ of latitude north and 81°1′ of longitude west and 19.01 m above sea level. The experimental phase was carried out on hydrated red ferralitic soil with medium fertility, moderately acidic (pH 5.60), low in phosphorus content (2.43 mg/100 g), and containing 0.18% total nitrogen, 3.2 % organic matter, calcium predominates (11.84 mEq/100 g) among the exchangeable cations and the cation exchange capacity was slightly low (19.21 mEq/100 g) as previously described (37). The climate of the region is tropical, seasonally humid, with an annual average temperature between 24.3°C and 33.4°C, and relative humidity of 80%. The annual rainfall is 1,331 mm, where 79.8% of it occurs between May and October (38).

**Figure 1:**
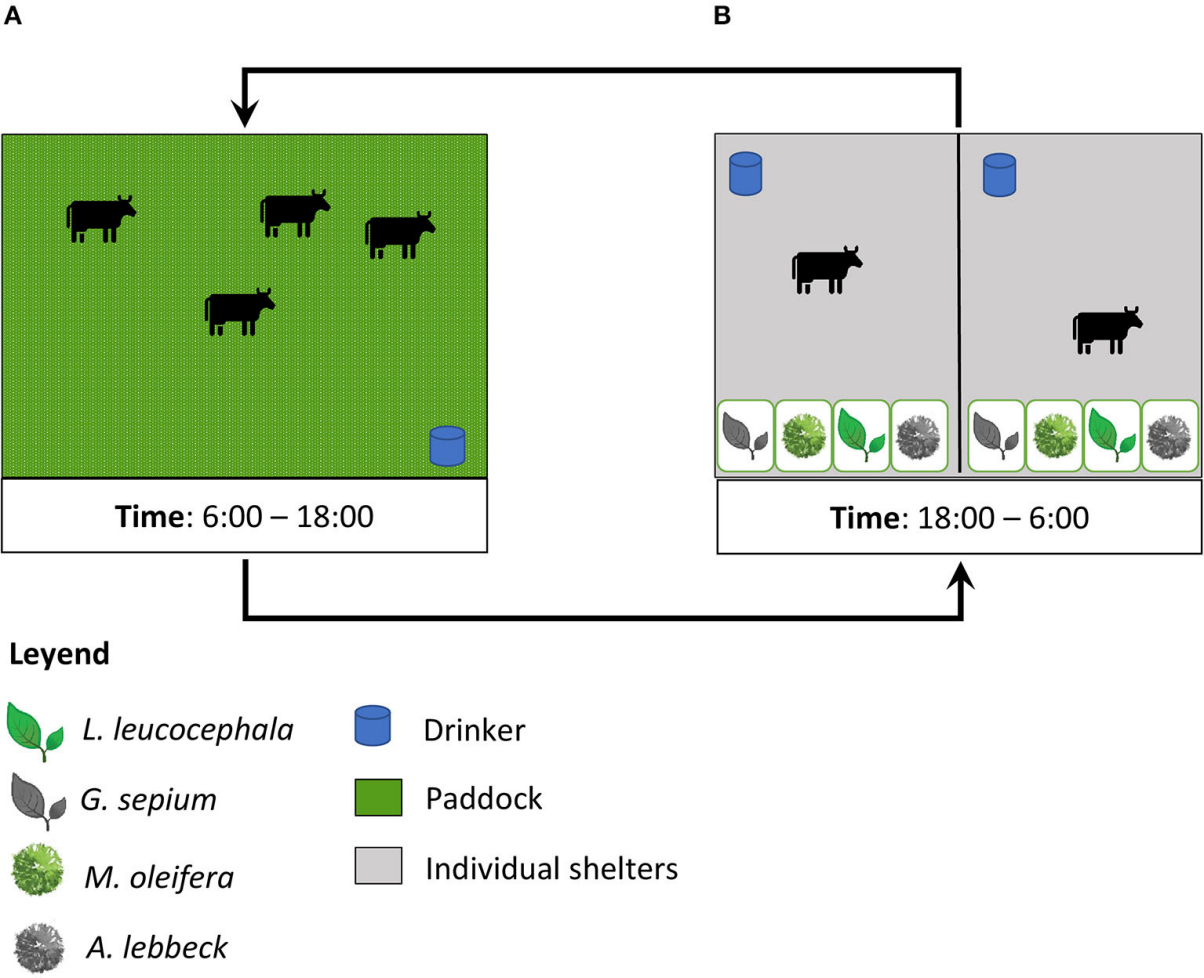
Description of management and experimental design. The figure shows the time animals spent in the pasture **(A)** and in the shelter **(B)**.

Twelve paddocks, each measuring 0.54 ha, were used through a rotation of 3 days of occupation and 42 days of rest. In the morning, the animals were moved to the paddocks, where the availability of feed was 2.45 Ton/ha of dry matter (DM), and from 18:00 h the animals had access to the leaves of the four species in the feeders (Figure 1). Grass availability was determined by the visual estimation method and the botanical composition was measured as previously described (6, 39). During the experiment, we collected five grass samples and one sample per tree leaf type. The feeding regime included the grazing of *Megathyrsus maximum* in paddocks and nocturnal supplementation with *Saccharum officinarum* (sugar cane) (0.52 kg DM/animal) and the PCN002 commercial feed for growing animals (MINAG, Habana, Cuba) (Table 1) (0.44 Kg DM/animal). In the grazing area (Figure 1A), free access to water for wallowing and natural shade by *Dichrostachys cinerea* (Marabú) was available in the wallowing areas. Drinking water and mineral salts were provided *ad libitum*.

**Table 1:** Chemical composition of ingredients in the diet.

	DM^*^ %	**CP (g/kg)**	**CF (g/kg)**	**GE Mcal/kg DM**	**Ca (g/kg)**	**P (g/kg)**
Chopped Sugar cane (3 mm) *S. officinarun*	26	28	240	2.19	0.6	0.1
Comercial feed	88	110	430	2.55	1.7	0.2
*M. maximus*	23	80	180	1.81	1.2	0.1
*L. leucocephala*	26.02	205	282	2.25	2.3	2.5
*A. lebbeck*	32.68	236	327.6	2.41	0.88	1.49
*G. sepium*	28.79	246	283	2.53	1.7	2.1
*M. Oleifera*	28.7	265.6	324.8	1.63	0.44	2.59

* *Dry matter (DM %), crude protein (CP, g/ KgDM), crude fiber (CF, g/ kgDM), Gross Energy (gross energy, Mcal/kgDM), calcium (Ca, g/kg DM), and phosphorous (P, g/kg DM)*.

### Experimental Design

In this study, the preference and consumption of fresh tree leaves were determined in a group of water buffaloes. *Leucaena leucocephala, Albizia lebbeck, Gliricidia sepium*, and *Moringa oleifera* were offered simultaneously (1.2 kg/animal of each fresh leaf type) to the animals. The buffaloes had no previous consumption experiences of the leguminous tree species studied. The trees were pruned and only the leaves that sprouted after 60 days were used in the experiment. Leaves of the trees were cut at a height of 1.7 meters, and the pods and stems were excluded. Fresh leaves were collected daily between the hours of 9:00–11:00. The trees were not irrigated nor treated with agrochemicals.

The animals were fed for 15 days with tree leaves and a base diet (Table 1). Experimental measurements were made daily from day 16 to day 32. After returning from the paddock, the animals were separated into individual shelters and each type of leaf was randomly placed separately in one of four feeders (each feeder measured 50 × 90 × 30 cm).

We used the repeated measured model shown in equation (1) below:

**Y_ijklm_ = m + T_i_ + P_j_ + A_j_ + e_ijklm_**

where:

Y_ijklm_ = represented the m-n measurement made in the l-n square the j-n period in the i-n treatment in the k-n sampling within the j-n period.

m = population means or general intercept.

T_i_ = fixed effect of the i-n treatment (i = 1: Ll, 2: Al, 3: Gs, 4: Mo).

P_j_ = fixed effect of the i-n day (i = 1,…. 16).

A_i_ = fixed effect of the i-n animal (i = 1, 2, 3.9)

e_ijklm_ = random residual associated with the m- n measurement. ~ N (0, s2).

### Analysis of Consumption and Chemical Composition of Fodder

The consumption of each leaf type was measured by subtracting the amount left in the feeders from the initial amount of leaves placed. For the weighing of fresh leaves, a digital scale with a sensitivity of 0.01 kg was used. Laboratory analysis was carried out at the beginning of the experiment to determine the DM, crude protein (CP), crude fiber (CF), and gross energy (GE). All procedures of feed analysis were performed as described in the manual “Official methods of analysis of AOAC international” (40).

The DM content of the leaves was determined individually in the laboratory of the Indio Hatuey Grass and Forage Experimental Station as described previously (41). To estimate the DM content, 500 g of homogenized grass samples were dried in a forced-air oven at 60°C for 48 h. The weight of each leaf sample was determined before and after the dehydration process (40) using an analytical balance of sensitivity 0.001 kg. After dehydration, the leaves were ground to a size of 2 mm and stored in amber glass jars.

The CP was calculated by the Kjeldahl method (42). CF was estimated by the Van Soest method (43, 44) briefly described by Garcia et al. (17). The GE was calculated with calorimetric methods using benzoic acid as the internal standard (45). This analytical procedure was performed with an adiabatic calorimetric pump (Model 1341EB, Parr instrument, Illinois, United States).

### Statistical Analysis

The results were analyzed using the SPSS program version 22 (IBM Corp, New York, United States). An analysis of variance (ANOVA) was applied to determine whether there were any statistically significant differences between the measurements. Each buffalo was considered as an experimental unit (9) during the experimental period of 16 days. The weight of the feed (leaves and other supplements) before and after its placement in the feeders was evaluated individually. A Pearson correlation test was performed for the consumption of dry matter for the four leaf types.

## Results

### Water Buffaloes Leaf Type Preferences

**Figure 2:**
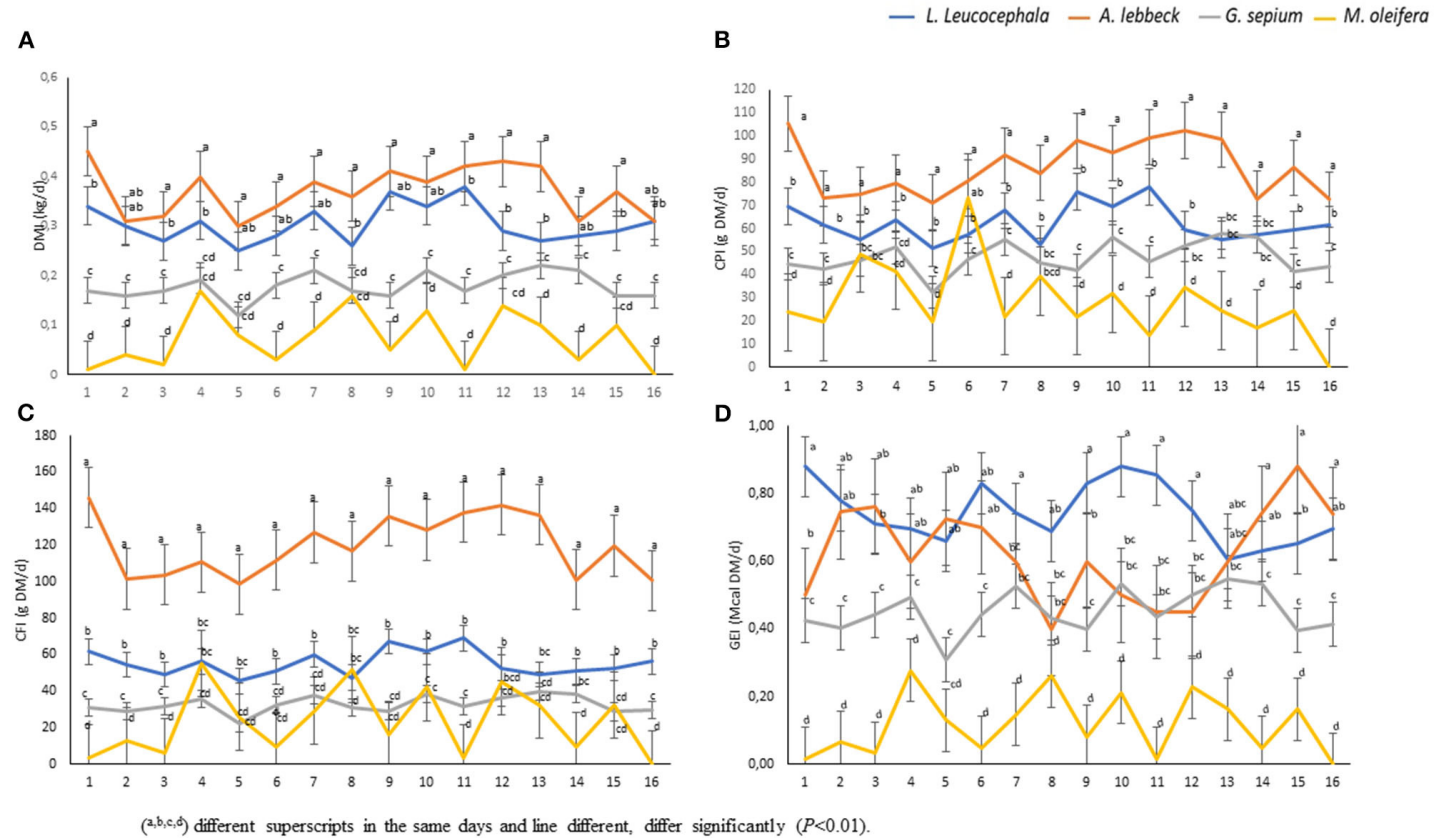
Consumption and overall nutritional contribution of the leaves. The statistical differences in the consumption of dry matter of the leaves are shown, as dry matter intake (DMI) **(A)**; crude protein intake (CPI) **(B)**; crude fiber intake (CFI) **(C)**; and gross energy intake (GEI) **(D)**. (Duncan, *P* < 0.01). *N* = 9 during 16 days of evaluation (*P* < 0.01).

**Figure 3:**
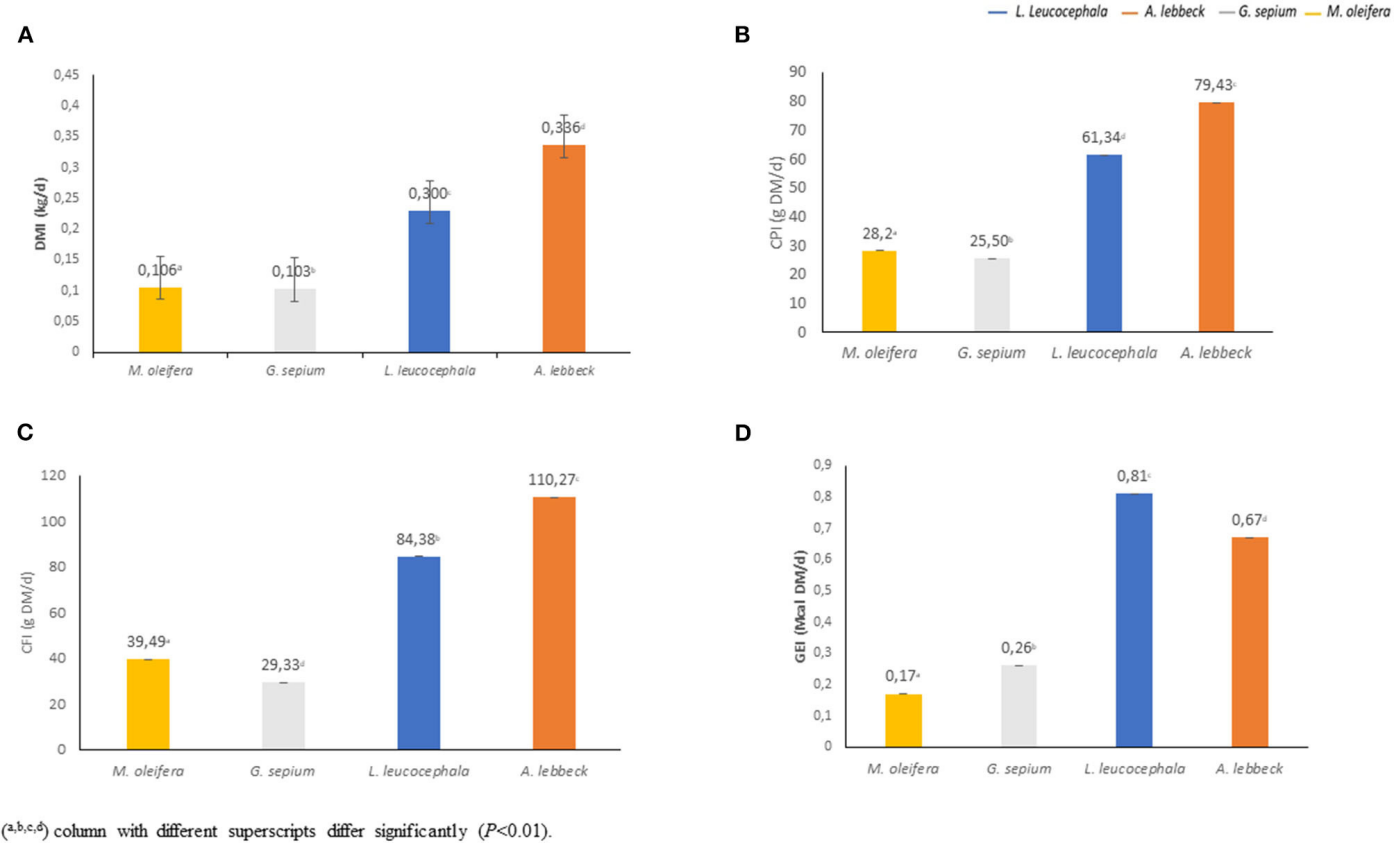
Nutritional contribution of each leaf type. The figure represents the average contribution (per animal/day) of dry matter intake (DMI) **(A)**, crude protein intake (CPI) **(B)**, crude fiber intake (CFI) **(C)** and gross energy intake (GEI) **(D)** (*P* < 0.01).

There was a daily variation in the consumption of the four types of leaves studied (Figure 2A). The leaves of *A. lebbeck* (0.34 kgDM) and *L. leucocephala* (0.30 kgDM) were the most consumed, respectively, while the leaves of *M. oleifera* (0.11 kgDM) *and G. sepium* (0.10 kgDM) were the least consumed (Figures 2A, 3A). Each animal consumed 0.85 kg of DM of leaves daily (*P* < 0.01). The consumption of *A lebbeck* and *L. leucocephala* was always the most consumed, but it was observed that this amount reduced when the buffaloes consumed higher quantities of *G. sepium* and *M. oleifera*.

### Contribution of Legumes to the Buffalo Diet

The analysis of the chemical composition revealed differences between the evaluated species (Table 1). The DM content was lower in *M. oleifera* and *G. sepium*. Notably, *M. oleifera* had the lowest GE values. *L. leucocephala* and *M. oleifera* were the species with the lowest and highest CP, respectively. CF content was lower in *G. sepium* and *L. leucocephala* but higher in *M. ole*í*fera* and *A. lebbeck* (Table 1). The contribution of each of the species in terms of feed intake was different (Table 2 and Figures 2A–D). Of the leaves consumed, DM content differed between the four tree species during the period evaluated (Figure 2A). The CPI was highest in *A. lebbeck* and *L. leucocephala* followed by *M. oleifera* and *G. sepium* (*P* < 0.01; Figures 2B, 3B). The CFI was different between treatments. *A. lebbeck* had the highest CF content followed by *L. leucocephala, M. oleifera*, and *G. sepium*, respectively (*P* < 0.01; Figures 2C, 3C). However, the consumption of GE was highest in *A. lebbeck* followed by *L. leucocephala, G. sepium*, and *M. oleifera*, respectively (*P* < 0.01; Figures 2D, 3D). No differences were found in the consumption of calcium, phosphorous, and organic material present in the four types of leaves.

**Table 2:** Principal nutrient composition in the diet.

	Nutrient intake of the diet^*^							
	**Quantity(kg)**	**DMI (kg/d)**	**CPI (g DM/d)**	**CFI (g DM/d)**	**GEI (Mcal DM/d)**	**GEI (Mcal DM/kg)**	**CaI(g DM/d)**	**PI(g DM/d)**
Chopped Sugar cane (3mm) *S. officinarun*	2.00	0.52	14.56	124.80	1.14	2.19	0.31	0.05
Comercial feed	0.50	0.44	48.40	189.20	1.12	2.54	0.75	0.09
*M. Maximus*	38.37	8.83	706.01	1,588.52	15.97	1.80	10.59	0.88
Subtotal		9.79	768.97	1,902.52	18.23		11.65	1.02
%		92.05	79.81	88.04	90.47		90.61	36.98
*L. Leucocephala*	1.15	0.30	61.34	84.38	0.67	2.23	0.69	0.75
*A. lebbeck*	1.03	0.34	79.44	110.27	0.81	2.38	0.30	0.50
*G. Sepium*	0.36	0.10	25.50	29.33	0.26	2.60	0.18	0.22
*M. Oleifera*	0.37	0.11	28.20	34.49	0.17	1.54	0.05	0.28
Subtotal		0.85	194.48	258.48	1.92		1.21	1.74
%		7.95	20.19	11.96	9.53		9.39	63.02
Total	43.78	10.63	963.45	2,160.99	20.15		12.86	2.76

* *Quantity (kg), dry matter intake (DMI, Kg/d), crude protein intake (CPI, g DM/d), crude fiber intake (CFI, g DM/d), gross energy intake (GEI, Mcal DM/d), gross energy intake per kg(GEI, Mcal DM/kg) calcium intake (CaI, g DM/d), and phosphorous intake (PI, g DM/d)*.

The average intake of fresh leaves was 2.91 kg/day and represents a daily DM consumption of 0.85 kg in this study (Table 2). The CPI of leaves was 194.48 g /animal/d; CFI was 258.48 g/ animal/d and GEI was 1.92 Mcal/animal/d.

The total DM Intake (leaves + *M. maximum* + *S. officinarum* + commercial feed) in this study was 10.62 KgDM/animal corresponding to 2.83 % body weight, and 936.45 g of CPI/animal, CFI = 2,160.99 g/animal; GEI 20.15 Mcal /animal, Ca = 12,86 g/animal and phosphorous = 2,76 g/animal (Table 2).

The DMI between *L. leucocephala* and *A. lebbeck* showed significant correlation (Pearson *r* = 0.54, *P* < 0.01); DM consumption of *G. sepium* and *M. oleifera* showed a positive correlation (Pearson *r* = 0.26; *P* < 0.01). Notably, the correlation of DMI between *L. leucocephala* and *M. oleifera* was negative (Pearson *r* = −0.28; *P* < 0.01); *A. lebbeck* and *G. sepium* had a value of *r* = 0.32. DMI of the most preferred and least preferred legume leaves (*L. leucocephala* + *A. lebbeck* vs. *G. sepium* + *M. oleifera*) showed a negative correlation (Pearson *r* = −0.43; *P* < 0.01).

## Discussion

Consumption of the leguminous trees was higher in *L. leucocephala* but provided less DM, CP and CF when compared to *A. lebbeck*. In this study, *A. lebbeck* was the leaf most consumed on the basis of DM, except for GE, where *L. leucocephala* was the most consumed species in the diet.

Previously, an evaluation in the paddock was conducted on bovine browsing behavior in an arboretum of 60 tree species. The findings showed a preference for *L. leucocephala* and *A. lebbeck* in cattle (46). This evaluation was repeated with buffaloes resulting in a similar preference for *L. leucocephala* (24). According to García et al. (18), *M. oleifera* was a moderately-consumed plant. *A. lebbeck and L leucocephala* was the most consumed species, probably due to the ease of collecting these branches in the feeders. *L. leucocephala* has very small leaves which are easily licked from the bottom of the feeders while *A. lebbeck* and *G. sepium* are more resistant to this type of action (47). However, it was observed that the rate of wilting of *M. oleifera*, prevents its consumption by the animals. We could attribute the preference for *L. leucocephala* and *A. lebbeck* to the “feeding memory” (i.e., the tendency of animals to remember the information of feed for up to 3 years) effect, reported in sheep (48) and buffaloes (49). The difference in the consumption and acceptability of the tree leaves could be suggestive of the satisfaction of the needs of the appetite and not that of “hunger” (50, 51). Additionally, ruminants prefer leaf types as a supplement that rapidly provides the highest satiety level of nutrients (52, 53).

The DM content of the feed in ruminants is partially responsible for limiting its consumption and ingestion through short-term regulation along with the fast fermentation of the carbohydrate contents (10, 50). These results are indicative of the satisfaction of the animal's needs and coincide with what was reported by Mendez and Lima (54) who indicated that the voluntary consumption (expressed in the percentage of body weight) is 2.59–3.09%) in buffalos. Similar results were previously published by Paul and Lai (55).

Tree leaves (*L. leucocephala, A. lebbeck, G. sepium*, and *M. oleifera*) contributed 194.48 g DM to the daily CPI. This represents 20.19% of the protein even though the trees only provided 7.95% of the DM in the diet. The rest of the diet provided 768.97 g CPI/animal/day (79.81% of diet). Paul and Lai (55) reported a daily CPI of 298–310 g in the diet of female buffaloes with a similar body weight of 300–350 kg and daily weight gain of 250 g/day, while being fed with different proportions of feed in India. The value of the proportion of CPI due to the ingestion of tree leaves in this study was similar to a study in bovines in Cuba (21, 45, 56). Of the four types of leaves, *A. lebbeck* was the one that contributed the most to the protein needs of buffaloes and was sufficient to cover all the requirements of the animals used. Valenciaga et al. (25, 57) estimated in Cuba that a DM intake of 9.95 and 10.25 kg DM in buffaloes with 600 kg of BW was needed.

Delgado et al. (12) reported an apparent digestibility above 60% for trees such as *G. sepium* and *L. leucocephala* in similar conditions in Cuba. In this sense, Albores-Moreno et al. (58) reported high voluntary consumption in cattle (10.26–12.48 kg of D/animal/day) of legumes and found that the secondary metabolite content of plants did not interfere with the voluntary intake. Additionally, Barros-Rodríguez et al. (28) report that up to 50% of *L. leucocephala* can be included in the diet. These criteria could explain the favorable consumption in favor of *L. leucocephala* and *A. lebbeck*, in the same way, that it can be attributed to the digestibility as reported by Delgado et al. (12). However, they do not explain why *G. sepium* and *M. olifera* were scarcely consumed.

Notably, the lower intake of *M. oleifera* could be related to the effects of this plant on the ruminal flora, volatile fatty acids, and ruminal ammonium concentration (59). This legume is low in fiber with higher degradability, gas production, short chain fatty acids production, and lower methane emissions as compared to wheat straw.

An *in vitro* study with an *M. oleifera* leaf extract as an alternative to monensin in sheep diets, found similar effects on the ruminal parameters (ruminal degradability, ammonia concentrations, and gas production) (60). The lower consumption of *M. oleifera* could also depend on the concentration of anti-nutritional factors (condensed tannin, tannins which are precipitants of proteins, terpenoids, and total sterols) (18, 31) and the ability of this leaf type to wilt, rather than its nutritional value. Previous studies attributed the texture, anti-nutritional factors and the apparent digestibility (17, 18, 32, 48) to the speed with which the branches lose qualities after being cut (61). It could also be attributed to the vegetative state or age (60- days-old) in which the leaves of the plants were collected (62, 63), climatic conditions (13, 56), the animal category (48), the amount and nutritional composition of the tree resource (64), DM content and apparent digestibility (32, 65–67). However, there does not seem to be a direct relationship between the nutritional composition and the palatability of the leaves of leguminous trees, and the leaves of *L. leucocephala* and *A. lebbeck* may generate similar stimuli in animals, meaning they are consumed more.

Pearson's correlation was significantly negative between the two most consumed species (*L. leucocephala* and *A. lebbeck*) and the two least consumed (*G. sepium* and *M. ole*í*fera*). In this sense, there was no correspondence between what was reported by Santana-Perez et al. (47) regarding the low correlations of consumption of *A. lebbeck* (stems with irregular barks) compared to *L. leucocephala, G. sepium*, and *M. oleifera* (smooth stems), attributing it to the physical characteristics of the stems.

Analysis showed a negative correlation between *L. leucocephala* and *M. oleifera* in the DM intake, similar results were obtained previously (17, 18) where during grazing they observed *M. olifera* being rejected in comparison with *L. leucocephala* and other leguminous trees. Santana-Perez et al. (47) found differences in voluntary consumption in sheep and cattle, attributing it to the differences between the maximum diameters of the stems consumed. With *G. sepium, L. leucocephala, M. oleifera*, and *A. lebbeck* being the most consumed, respectively, the sheep did not consume the stems of *G. sepium*, attributing this phenomenon to the aversive stimulus generated by this plant.

## Conclusions

There were significant differences in the consumption and acceptability of the leaves of the tree species. The highest consumption of leaves was that of *L. leucocephala* and *A. lebbeck*, while *M. oleifera* and *G. sepium* species were consumed in lower quantities. The leaves of *A. lebbeck* contributed more nutritionally to the animals, although the amounts consumed were equal to *L. leucocephala*. This study helps to define which tree species could be used in production systems with buffaloes in the tropics by providing insights into buffaloe preferences for different leaves. We suggest that future studies explore the effects of secondary metabolites on feed intake.

## Data Availability Statement

The datasets generated for this study are available on request to the corresponding author.

## Ethics Statement

The animal study was reviewed and approved by the ethics committee of the Experimental Station: Indio Hatuey, Cuba.

## Author Contributions

All authors also participated equally in the design, preparation, and review of this research.

## Conflict of Interest

The authors declare that the research was conducted in the absence of any commercial or financial relationships that could be construed as a potential conflict of interest.
